# Association of Anxiety and Depression in Patients Undergoing Cardiac Catheterization With Number of Major Coronary Artery Stenosis: A Cross-Sectional Study

**DOI:** 10.7759/cureus.21630

**Published:** 2022-01-26

**Authors:** Dipak R Das, Mihir R Nayak, Debjyoti Mohapatra, Debasish Mahanta

**Affiliations:** 1 Cardiology, Srirama Chandra Bhanja (SCB) Medical College and Hospital, Cuttack, IND; 2 Psychiatry, Srirama Chandra Bhanja (SCB) Medical College and Hospital, Cuttack, IND; 3 Community Medicine, All India Institute of Medical Sciences (AIIMS), Bhubaneswar, IND; 4 Community Medicine, Srirama Chandra Bhanja (SCB) Medical College and Hospital, Cuttack, IND

**Keywords:** diabetes, hypertension, coronary angiogram, coronary artery disease, hospital anxiety depression scale (hads)

## Abstract

Introduction

The relationship between the level of anxiety and depression among coronary artery disease (CAD) patients and coronary angiographic findings is ambiguously mentioned in studies. Past evidence shows that the relationship between anxiety and depression with coronary artery disease can be bidirectional. There is a paucity of literature on the association of levels of anxiety and depression with the number of coronary arteries involved in coronary artery disease.

Methods

This study was conducted in a tertiary care hospital to find the level of anxiety and depression in patients undergoing cardiac catheterization and their association with the numbers of the major coronary artery involved. Patients undergoing cardiac catheterization in the Department of Cardiovascular Science of a tertiary care hospital in India from May 2020 to December 2020 were considered for inclusion in the study. Coronary artery disease was diagnosed based on the combination of clinical, ECG, echocardiography, or biomarker parameters in various combinations. These patients were further subjected to coronary angiogram to know the extent of stenosis and the number of coronary vessels involved in the disease. The level of anxiety and depression was measured by using the Hospital Anxiety and Depression Scale (HADS) during the period of admission and at least 24 hours after diagnosis and at least 12 hours before cardiac catheterization. The data was entered into SPSS software version 22.0 (Armonk, NY: IBM Corp.) for statistical analysis. Chi-square test, Kolmogorov-Smirnov test, and Kruskal-Wallis test were used for the interpretation of data and find an association between severity of anxiety and depression with the number of major coronary vessels involved.

Results

Anxiety was seen in 83.3% of the patients with 31.5% having severe anxiety. Depression was found in 77.8% with 38.9% suffering from severe depression. The anxiety and depression scores of HADS were significantly higher in those with triple-vessel disease compared to the double-vessel or single-vessel disease.

Conclusion

Screening and management of anxiety and depression is an essential part of the care of patients with coronary artery disease. People with triple-vessel disease need the most attention and appropriate management of anxiety and depression.

## Introduction

Anxiety and depression have been seen among hospitalized patients suffering from heart disease whether they are admitted in an emergency condition or through an outpatient unit. Chronic anxiety and depression have been shown to have an impact on cardiac health [1]. The myocardium’s response to emotions in its way makes it susceptible to physiological, and anatomical alternation [1].

Anxiety has shown to increase the psychological and physiological activities of the body such as heartbeat, blood pressure, and cardiac output, and this puts the cardiovascular system at risk. The increased sympathetic nervous system output increases the probability of the development of atherosclerosis and subsequent coronary artery disease (CAD) [2].

Various mechanisms like behavioral and lifestyle factors, sympathetic overactivity, increased platelet activation, and autoimmune and inflammatory mechanisms explain how depression increases the risk of CAD [3]. Elevated platelet serotonin levels, promoting clotting, may be associated with depression and the occurrence of CAD [4]. Patients with depression have been shown to have elevated levels of C-reactive protein (CRP) and pro-inflammatory cytokines, such as tissue necrosis factor, interleukin-1, interleukin-2, and interleukin-6 [4]. It has been postulated that inflammation causes an increase in atherosclerosis thus increasing the risk of CAD [4].

Both anxiety and depression may play a significant role in the development of various cardiac problems including coronary artery disease. But this relationship can be bidirectional [5]. Studies from India and other places have shown a high prevalence of psychiatric disorders, particularly anxiety and depression in CAD patients [6,7]. Also, depression and anxiety have been found to worsen prognosis and quality of life in patients with CAD [3]. Psychological problems contribute to the development of cardiovascular disease and also develop as a complication of it [4]. The person may have chronic anxiety or depressive symptoms over time without meeting the clinical criteria for anxiety or depressive disorder respectively [3]. There are chances that mere knowledge of suffering from coronary artery disease may affect the mental health of an individual. In India where mental health is a subdued topic, screening for patients for anxiety and depression when they present with features of coronary disease may be a late but beneficial intervention.

The association of the level of anxiety and depression with the number of major coronary arteries involved may have a bearing on the treatment modality of such patients. In this regard, the current study was done to find the level of anxiety and depression in patients undergoing cardiac catheterization and their association with the numbers of major coronary arteries involved in stenosis.

## Materials and methods

This was a hospital-based, cross-sectional study, conducted in the Department of Cardiovascular Science in collaboration with the Department of Psychiatry of Sriram Chandra Bhanja Medical College and Hospital, Cuttack, Odisha. This is the largest tertiary care government hospital in Odisha, an eastern region state of India. The study was conducted over eight months from May 2020 to December 2020. Institutional review board approval was obtained from the institutional ethics committee (IEC) of SCB Medical College and Hospital, Cuttack (approval #192), before the initiation of the study. The study had to be completed within a stipulated time frame. All the patients who had come to the hospital during the study period and fulfilled our inclusion criteria were considered eligible for inclusion.

Inclusion and exclusion criteria

Patients aged between 30 years and above who were admitted to the cardiology unit during the study time period and were newly diagnosed with coronary artery disease with more than 70% blockage and had been scheduled for cardiac catheterization were eligible for inclusion in the study. Also, patients previously diagnosed with psychiatric illnesses other than depression and anxiety disorder, patients undergoing emergency cardiac catheterization or within 24 hours of diagnosis, and patients with insignificant (non-obstructing) luminal blocks of <70% were excluded from the study.

The diagnosis of CAD was made by a cardiologist based on clinical, ECG, echocardiography, or biomarker parameters in various combinations. Further, these patients were subjected to coronary angiography to know the extent of coronary artery stenosis and the number of vessels involved. Coronary angiogram (CAG) reports of ≥70% artery luminal diameter obstruction in at least one of the major coronary arteries were considered significant [8]. Based on the CAG report, patients were classified into a single-vessel, double-vessel, or triple-vessel disease.

Patients and caregivers were explained in the regional language in detail about the study procedure. Written informed consent for participation in the study was taken from the enrolled patients. The researcher had designed a semi-structured interview schedule was used to capture the socio-demographic and clinical history of patients. The level of anxiety and depression was measured by the researchers by using the Hospital Anxiety and Depression Scale (HADS). HADS was administered during the period of admission at the cardiology ward for elective cardiac catheterization and at least 24 hours after diagnosis and at least 12 hours before cardiac catheterization. Based on the HADS scores subjects were classified as follows: normal (score: 0-7), mild (score: 8-10), moderate (score: 11-14), and severe (score: 15-21) [9].

Statistical analysis

Collected data were entered into excel sheets. The data was cleaned and analyzed using SPSS software version 22.0 (Armonk, NY: IBM Corp.). Descriptive statistics were used for the analysis of socio-demographic and clinical variables. Chi-square test was done to test the association between socio-demographic variables and the presence of co-morbidities with the three categories of CAD. The Kolmogorov-Smirnov test was used to check the normal distribution of scores of anxiety and depression. The scores across the three groups were not normal in distribution. Kruskal-Wallis test was used to compare the median HADS across the three groups. A p-value of <0.05 was considered as a significant result.

## Results

A total of 73 patients were screened for inclusion in our study. Among them, 13 participants had an insignificant luminal block (<70%), three had a history of psychiatric illness, and three patients who refused consent were excluded from the study. A total of 54 patients were finally included in the study. Based on the number of vessels involved in the coronary angiogram, patients were initially classified as a single-vessel disease (SVD), double-vessel disease (DVD), or triple-vessel disease (TVD). The number of patients in each group and their socio-demographic and clinical characteristics are provided in Table 1. Except for education status, there was no significant association of any of the socio-demographic status with the number of coronary vessels involved. The proportion of patients having triple-vessel disease gradually increased as the education status increased. This study did not find any significant association with any of the existing co-morbid conditions like hypertension, diabetes mellitus, or dyslipidemia with the number of coronary vessels involved. Diabetes mellitus was the most common co-morbid condition in our study with more than 50% having the disease. Smoking was more common compared to the harmful use of alcohol in this study. But neither smoking nor harmful use of alcohol was associated with the number of coronary arteries involved.

**Table 1:** Socio-demographic and clinical characteristics and their association with numbers of coronary vessels involved.

Variables		Single-Vessel Disease (SVD), n=21 (39%)	Double-Vessel Disease (DVD), n=18 (33%)	Triple-Vessel Disease (TVD), n=15 (28%)	Total (%)	p-Value
Gender	Male	9 (32.1)	12 (42.9)	7 (25)	28 (100)	0.292
	Female	12 (46.1)	6 (23.1)	8 (30.8)	26 (100)	
	Mean age	59.14+9.18	58.61+6.75	60.33+8.78	59.3+8.19	0.835
Domicile	Rural	18 (43.9)	13 (31.7)	10 (24.4%)	41 (100%)	0.379
	Urban	3 (23.1%)	5 (38.5%)	5 (38.5%)	13 (100%)	
Educational level	Illiterate	4 (80%)	0	1 (20.0%)	5 (100%)	0.012
	Primary	13 (59%)	4 (18.1%)	5 (22.7%)	22 (100%)	
	Secondary	3 (18.6%)	9 (56.3%)	4 (25.0 %)	16 (100%)	
	Graduate	1 (9.1%)	5 (45.4%)	5 (45.4%)	11 (100%)	
Diabetes mellitus	Present	12 (42.8%)	8 (28.6%)	8 (28.6%)	28 (100%)	0.725
	Absent	9 (34.6%)	10 (38.5%)	7 (26.9%)	26 (100%)	
Hypertension	Present	7 (36.8%)	6 (31.6%)	6 (31.6%)	19 (100%)	0.9
	Absent	14 (40%)	12 (34.3%)	9 (25.7%)	35 (100%)	
Dyslipidaemia	Present	9 (50%)	6 (33.3%)	3 (16.7%)	18 (100%)	0.358
	Absent	12 (33.3%)	12 (33.0%)	12 (33.3%)	36 (100%)	
Alcohol	Yes	1 (11.1%)	5 (55.5%)	3 (20.0%)	9 (100%)	0.145
	No	20 (44.4%)	13 (28.9%)	12 (26.7%)	45 (100%)	
Smoking	Yes	4 (30.8%)	5 (38.4%)	4 (30.8%)	13 (100%)	0.786
	No	17 (41.5%)	13 (31.7%)	11 (26.8%)	41 (100%)	

Table 2 shows the HADS scores of anxiety and depression of patients enrolled in our study. A total of 83.3% of the patients had anxiety with 31.5% having severe anxiety; 77.8% of the patients had depression with more than half of them (38.9%) having severe depression.

**Table 2:** Distribution of study participants according to HADS score. HADS: Hospital Anxiety and Depression Scale

Variables	Normal (HADS: 0-7)	Mild (HADS: 8-10)	Moderate (HADS: 11-14)	Severe (HADS: 15-21)	Total
Anxiety (no. of patient, %)	9 (16.7%)	15 (27.8%)	13 (24.0%)	17 (31.5%)	54 (100%)
Depression (no. of patient, %)	12 (22.2%)	12 (22.2%)	9 (16.7%)	21 (38.9%)	54 (100%)

The median HADS score of anxiety and depression and their association with the number of vessels involved in CAD are shown in Table 3. In this study, we compared the median scores of anxiety and depression across the three groups and found that there was a significant difference between the groups.

**Table 3:** Median HADS score and its association with the number of vessels involved in CAD. HADS: Hospital Anxiety and Depression Scale; CAD: coronary artery disease

Variables	Single-Vessel Disease (SVD) (n=21)	Double-Vessel Disease (DVD) (n=18)	Triple-Vessel Disease (TVD) (n=15)	p-Value
Median score for anxiety (range)	9 (1-14)	10 (1-18)	17 (11-20)	<0.001
Median score for depression (range)	7 (0-20)	14 (3-21)	19 (14-21)	<0.001

We also carried out a post hoc analysis of the HADS score across the three categories of CAD to know about the difference between the pair-wise score of anxiety and depression. The median score of anxiety in triple-vessel disease was 17 which was significantly more than the scores of double-vessel disease (p<0.001) and single-vessel disease (p<0.001). There was no significant difference between single-vessel disease and double-vessel disease (p=0.346). The Depression score also significantly varied between the three groups. Depression score in those with the triple-vessel disease was significantly more compared to those with double-vessel disease (p=0.03) or single-vessel disease (p=0.01). There was also a significant difference in the depression score of single-vessel disease and double-vessel disease (p=0.02).

## Discussion

This study was conducted in the largest tertiary care government hospital in the state of Odisha, India. This hospital caters to the health need of the entire state. Of 73 people screened for inclusion in our study, 13 of them had an insignificant luminal block (<70%), three had a previously known psychiatric illness, and three patients refused to give consent and were thus excluded from the study. Thus, finally, 54 patients enrolled in the study. The numbers of patients who fall in SVD, DVD, and TVD groups were 21 (39%), 18 (33%), and 15 (28%), respectively. Males comprised slightly more than half of the patients in the study. Global burden of disease study has shown absolute burden, mortality, and disability-adjusted life-years lost to be more in males compared to females in India [10]. The present study was a hospital-based study where patients would have come only after developing a more debilitating disease. Women in India have been known to be neglected on the health front. However, recent trends have suggested an increasing burden of ischemic heart disease among females in India [11]. This might account for an almost equal distribution of both genders in our study, although it may not represent the actual community-based situation in India. This study did not show any significant difference across gender with respect to the number of vessels involved in coronary artery disease. The mean age of patients in this study was found to be 59.3±8.19 years. There was no significant difference in age across the three groups of patients. CAD is also more prevalent in the elderly and the peak period is attained between 51 and 60 years [12]. In India, the pattern of CAD has been reported to be a decade earlier compared with the age of incidence in developed countries [12]. The majority of subjects in the present study were from rural backgrounds. In the Indian context, the majority of people stay in the rural area, which is reflected as the higher rural population in the study sample. A patient from a rural area having lower socio-economic status prefers to go to a government setup where the expenditure for treatment is minimal as government bears the cost of treatment. The government tertiary teaching hospital stands as a good option for such patients.

Among socio-demographic status, only education status showed a significant association with the number of coronary vessels involved in CAD. Triple-vessel disease proportion was maximum in those with graduate or secondary education. Educational status was inversely proportional to coronary artery disease in a study conducted among the Chinese population [13]. In another study done by Gupta et al. among Indians, risk factors for cardiovascular diseases were significantly more in those with low education status [14]. Literature about the association between the number of coronary vessels involved and education status is scant and the present study is one of the pioneer studies exploring this association.

Diabetes was the most common co-morbid condition seen in CAD patients with more than 50% of patients having diabetes mellitus. Dyslipidemia and hypertension were seen in around one-third of study participants. In a study conducted in the Netherlands among patients attending primary health care, almost a quarter of male and female participants with coronary vascular disease had diabetes [15]. The higher prevalence of diabetes in India might be accounting for the higher prevalence of diabetes in the present study. In a study conducted in an urban area of India, about 21% of diabetic patients suffered from coronary vascular disease and more than one-third had either diabetes or impaired glucose tolerance combined [16]. The above study was done in 2001, and there has been a rise in diabetic cases in the last two decades. The INTER-HEART study conducted across a wide number of countries also found diabetes and hypertension to be risk factors for coronary artery disease [17]. This study did not find any significant association between the number of vessels involved in CAD and the presence of other comorbidities like diabetes, hypertension, and dyslipidemia.

Tobacco use was seen in almost a quarter of study participants with coronary vascular disease. But there was no association between the number of coronary vessels involved and tobacco use. Our sample size was too small to be powered enough to detect any such difference and a larger sample size could have given more insight.

This study found 83.3% of coronary artery disease patients with some form of anxiety. Severe anxiety was seen in 31.5% of patients. Median anxiety score was significantly more in those with the triple-vessel disease compared to those with double-vessel disease or single-vessel disease. No such significant difference was seen between single-vessel disease and double-vessel disease.

Assari et al. had found an inverse association between anxiety and the number of vessels involved in coronary artery stenosis [18]. The level and extent of stenosis are multi-factorial and other factors might explain the difference from this study. In another study done by Delewi et al. in the European population, anxiety scores were considerably higher at the pre-procedural level compared to the post-procedural level [19]. In the present study, an evaluation of anxiety was done at the pre-procedural stage. Post-procedural level anxiety has not been studied in the present study. In another study done exclusively among women, anxiety was inversely associated with the extent of coronary artery occlusion [20]. It is possible that in western countries coronary screening is common among people with anxiety who show other somatic symptoms associated with anxiety [20]. Low anxiety people might be referred later for screening and the extent of coronary occlusion might have increased at that time. In the present study, we have measured anxiety in patients who presented with coronary symptoms. High anxiety scores are independently associated with high mortality and non-fatal myocardial infarction in patients with coronary artery disease [21].

A meta-analysis of 20 studies done in 2010 showed that the presence of anxiety at baseline was associated with a 26% increase in the risk of the onset of heart disease and a 48% increase in cardiac death in a non-psychiatric sample over a mean follow-up period of 11.2 years [22]. Janszky et. al. had also found a similar association between anxiety and coronary heart disease [23]. The present study was a cross-sectional study and it is difficult to comment whether anxiety was a precursor or developed following the diagnosis of coronary artery disease.

Depression was also seen in almost 77% of cases, with severe depression seen in close to 39% of patients. The median scores of depression were significantly different in all three groups. It was highest in those with triple-vessel disease. The median score of depression in those with double-vessel disease was also significantly more compared to those with single-vessel disease.

Depression and coronary artery disease are known to have a bidirectional association [24]. Numerous mechanisms have been suggested for the association between clinical depression and heart disease [25]. In a study done by Ford et al. where medical students were followed up for 40 years, clinical depression was found to be an independent risk factor for depression [26]. A meta-analysis done by Gan et al. has established depression to be a risk factor for coronary heart disease and myocardial infarction [27].

A study conducted by Allabadi et al. among Palestinian patients scheduled to undergo cardiac catheterization shows that 54% of the patients had severe depression (cardiac depression score {CDS}>100) and 19.2% had severe to very severe anxiety (depression anxiety stress scale {DASS} score>15) [4]. In this study, almost 40% had severe depression. In the present study depression score in triple-vessel disease was significantly more than those with double-vessel disease or single-vessel disease. A study done among the Swedish population had shown people with moderate depression to be at higher risk of ischemic or hypertensive disease compared to those with mild or severe depression [28]. The study tool used to assess depression was a major depression inventory (MDI). The above study was longitudinal and was done through a questionnaire format among participants in a community-based study. The present study was done in a hospital setting by using a different tool. This may be the reason for different findings in our study.

The EUROASPIRE III study showed the prevalence of depression among patients with coronary artery disease varied from 8.2% to 35.7% in men and from 10.3% to 62.5% in women. Prevalence of anxiety varied from 12.0% to 41.8% in men and from 21.5% to 63.7% in women [29]. The assessment of depression and anxiety in the EUROASPIRE III study was done after six months after coronary heart disease diagnosis. Davidson et al. showed that optimizing treatment for depression cannot only cause a significant reduction in depressive symptoms but also improve cardiac prognosis [30]. In the present study, all the patients irrespective of their anxiety and depression status made an uneventful recovery. There was no follow-up of patients in the present study and thus we cannot comment on the future prognosis of such patients and if it has any association with the severity of depression and anxiety. It is evident from the current study that anxiety and depression are high in patients with more vessels involved. There is a need of integrating mental health care into CAD management.

Limitations of the study

Cross-sectional nature of the study limits assessment of the long-term impact of anxiety and depression on CAD. Patients with normal coronary angiogram or non-obstructing CAD findings were not included in the study which would have enabled us to make a better comparison. The concurrent COVID-19 pandemic could have affected the anxiety and depression levels of selected participants. The study had to be completed within a stipulated time frame and enrollment was restricted to all eligible patients providing consent during the stipulated study duration. A sample size of 54 was small to generalize the findings of the study.

## Conclusions

It is evident from the current study that anxiety and depression levels have a significant association with the number of coronary arteries involved in CAD. Patients with triple-vessel disease are likely to have high levels of anxiety and depression. Anxiety and depression should be screened in all patients reporting coronary artery disease and appropriately managed. Cognitive-behavioral therapy (CBT), stress reduction technique, and pharmacotherapy may be considered in patients with CAD having anxiety or depression. A follow-up strategy may be planned for such patients at intervals. In India, where mental health problems are stigmatized to date, the integration of mental health care with cardiac care may have a positive impact on the management of CAD.
